# Collaborating with health economists to advance implementation science: a qualitative study

**DOI:** 10.1186/s43058-020-00074-w

**Published:** 2020-09-29

**Authors:** Miya L. Barnett, Alex R. Dopp, Corinna Klein, Susan L. Ettner, Byron J. Powell, Lisa Saldana

**Affiliations:** 1 Department of Counseling, Clinical, & School Psychology, University of California, Santa Barbara, Santa Barbara, California, 93106-9490 USA; 2 Department of Behavioral and Policy Sciences, RAND Corporation, 1776 Main Street, Santa Monica, CA 90401 USA; 3 Division of General Internal Medicine and Health Services Research, Department of Medicine, David Geffen School of Medicine, University of California, Los Angeles, Los Angeles, CA 90095 USA; 4 Department of Health Policy and Management, Fielding School of Public Health, University of California, Los Angeles, Los Angeles, CA 90095 USA; 5 Brown School, Washington University in St. Louis, One Brookings Drive, Campus Box 1196, St. Louis, MO 63130 USA; 6 Oregon Social Learning Center, 10 Shelton McMurphey Blvd, Eugene, OR 97401 USA

**Keywords:** Health economics, Economic evaluation, Team science

## Abstract

**Background:**

Implementation research infrequently addresses economic factors, despite the importance of understanding the costs of implementing evidence-based practices (EBPs). Though partnerships with health economists have the potential to increase attention to economic factors within implementation science, barriers to forming these collaborations have been noted. This study investigated the experiences of health economists and implementation researchers who have partnered across disciplines to inform strategies to increase such collaborations.

**Methods:**

A purposeful sampling approach was used to identify eight health economists and eight implementation researchers with experience participating in cross-disciplinary research. We used semi-structured interviews to gather information about participants’ experiences with collaborative research. Thematic analysis was conducted to identify core themes related to facilitators and barriers to collaborations.

**Results:**

Health economists and implementation researchers voiced different perspectives on collaborative research, highlighting the importance of increasing cross-disciplinary understanding. Implementation researchers described a need to measure costs in implementation studies, whereas many health economists described that they seek to collaborate on projects that extend beyond conducting cost analyses. Researchers in both disciplines articulated motivations for collaborative research and identified strategies that promote successful collaboration, with varying degrees of convergence across these themes. Shared motivations included improving methodological rigor of research and making a real-world impact. Strategies to improve collaboration included starting partnerships early in the study design period, having a shared interest, and including health economists in the larger scope of the research.

**Conclusions:**

Health economists and implementation researchers both conduct research with significant policy implications and have the potential to inform one another’s work in ways that might more rapidly advance the uptake of EBPs. Collaborative research between health economists and implementation science has the potential to advance the field; however, researchers will need to work to bridge disciplinary differences. By beginning to develop strong working relationships; increasing their understanding of one another’s disciplinary culture, methodology, and language; and increasing the role economists have within research design and execution, both implementation researchers and health economists can support successful collaborations and robust and informative research.

Contributions to the literature

- Increased collaborations between implementation researchers and health economists have been called for to address the paucity of economic evaluations within implementation science.
- We found that these collaborations might be limited by differing disciplinary cultures and goals, especially around the measurement of implementation costs.
- Findings inform recommendations to increase and improve collaborations between health economists and implementation scientists to advance shared goals around conducting methodologically rigorous research that impacts real-world issues related to the uptake and sustainment of EBPs.

## Background

Stakeholders have identified costs and economic impacts as key factors that drive decision-making about implementing EBPs [1, 2]. Not only do decision makers need to know the cost of an EBP, but also it is important to understand the cost of the implementation process itself. Under- or over-estimating the costs of implementation could lead to failed implementation. Decision makers might not adopt a practice if they believe it will be too expensive. Likewise, if an implementation effort is not adequately financed, it is unlikely to be sustained [2]. Although cost has been identified as a key implementation outcome, it is not frequently evaluated within implementation studies [2–5]. In order for decision makers to determine if they are going to implement an EBP and the associated strategies to implement it, information on cost is needed. Limited research has compared the relative costs and benefits of different implementation strategies; increasing economic evaluations has been identified as a top priority for enhancing the public health impact of implementation strategies [4–6]. To accomplish this goal, increased collaborations between the fields of health economics and implementation science have been recommended to improve the economic evidence related to implementation strategies [5].

Economic evaluations within implementation science have predominately consider the economic impacts (relative to effectiveness) of (a) EBPs as implemented in community settings and, more recently, (b) implementation strategies used to support the implementation of EBPs [3–5]. Such research provides critical information for health system administrators, policymakers, payers, and provider organization leaders, who must make decisions about when and how to invest in EBPs [1]. Traditional methods of economic evaluation in health care (e.g., cost-effectiveness and benefit-cost analysis [7]) compare incremental differences in costs and outcomes among discrete and highly specified clinical practices. These methods have required adaptation for use in implementation studies, with a focus on different types of costs and outcomes, lessening of experimental control, and a health system/infrastructure (rather than health care provider) point of view [8].

Despite these methodological advances, economic evaluations remain rare in implementation research—let alone other types of research examining economic factors (e.g., influence of market forces on supply of and demand for EBPs) or financing (e.g., sustainable approaches to funding various training and quality assurance activities). Various reasons contribute to the paucity of economic evaluations in implementation science, including difficulty tracking implementation costs, inconsistent resource needs given the varying stages in which organizations are positioned at the time of adopting a new practice, and reluctance to disclose financial information. Furthermore, though implementation researchers come from a variety of disciplines, the majority of identified leaders in the field tend to have backgrounds in social work, psychology, and public health, with limited representation from health economics [9–11].

A recent review of health economic evaluations in implementation and quality improvement science called for “increased collaboration between health economics and implementation science” in order to address gaps in the literature [5]. Therefore, one relatively efficient and impactful strategy to improve economic evaluation in implementation science is to collaborate with health economists as part of cross-disciplinary teams. Health economists are well-positioned to contribute to implementation research as their work investigates health care resource allocation, efficiency, and value as well as consumer, practitioner, and healthcare system behaviors [12]. Cross-disciplinary collaborations can be characterized as being multidisciplinary, interdisciplinary, or transdisciplinary, which range in their level of integration across disciplines [13, 14]. In multidisciplinary teams, researchers work within their own disciplines on a common research problem, with the goal to eventually combine efforts, whereas researchers in interdisciplinary teams are more interactive throughout the research project, with researchers each drawing from their own disciplines. Transdisciplinary research is the most collaborative as it spans disciplinary bounds to develop new scientific frameworks and methodologies [13]. Aarons and colleagues [15] have identified cross-disciplinary dissemination and implementation science teams as a way “to expedite the translation process through having the most relevant and innovative perspectives come together to determine the most efficient and effective ways to move research to practice (p.2).” Yet, challenges exist with how to best identify and partner with health economists who are interested in typical implementation research. There are documented shortages of individuals with health economics expertise to consult on grants [16]. Adding to the challenge, health economists often are contacted late in a project, rather than being consulted early on to plan for appropriate research design [16]. Consistently, economic evaluations have been treated as adjunctive to the main study design, which can be dropped if there is not adequate grant funding [17].

Although implementation science lends itself to transdisciplinary work by its nature, a recent survey of dissemination and implementation researchers found a tendency for researchers to collaborate with individuals from within their own discipline (e.g., social work or psychology) [11]. Furthermore, the perspectives of health economists have not been included in past research on team science in dissemination and implementation research [15, 18]. Given calls to increase economic evaluations within implementation research, the potential of collaborating with health economists, and the documented challenges in doing so, further understanding is needed on how to best facilitate these collaborations. In this study, we explore the experiences of health economists and implementation researchers from other fields (e.g., psychology) who have conducted collaborative implementation research involving economic evaluations. The purpose of this study is to understand the challenges associated with partnerships between health economists and implementation researchers and to inform future collaborative efforts.

## Methods

### Design

Expert interviews were conducted with health economists and implementation researchers who had experience conducting collaborative research on topics related to implementation science and health economics. Purposeful sampling was conducted, using a combined criterion-i and snowball sampling approach [19]. First, using a criterion-i sampling strategy, which is a purposeful sampling strategy where participants meet a predetermined criterion of importance to the study, members of the research team (M.B., A.D., L.S., and B.P.) identified researchers with experiences (i.e., existing publications or grants related to implementation research) collaborating across the two fields. Second, at the end of every interview, participants were asked if they knew other researchers from either field who had conducted research in the area of implementation research and health economics. With the use of snowball sampling, some implementation researchers and health economists had collaborated on projects together, but the majority had not or had collaborated with a range of researchers across the two disciplines, which informed their interview responses. Given the low-risk nature of the study, and the lack of personal information obtained in the interviews, the protocol received an exemption from the University of California, Santa Barbara’s Institutional Review Board. We described the methods, results, and interpretations consistent with the Consolidated Criteria for Reporting Qualitative Research [20], included in Additional file 1.

### Participants

Identified researchers across the two disciplines were emailed by the interviewers, who described the purpose of the study and inquired if the researcher would be willing to participate in an expert interview. Of the researchers contacted, 61.5% of the health economists and 72.7% of the implementation researchers agreed to participate in the study (*n* invited = 13 and 11 respectively). Those who did not participate either did not respond to email requests or stated that they were too busy. Eight health economists and eight implementation researchers from psychology, public health, and engineering (hereafter referred to as implementation researchers) participated in interviews. Health economists and implementation researchers were employed in a variety of settings, including academic institutions, the US Department of Veterans Affairs, and policy centers. They had varied research interests, ranging from substance use and behavioral health disorders to chronic medical conditions. Each of them had experience collaborating across disciplines in the evaluation of interventions or the implementation of interventions, though this experience varied. Implementation researchers ranged in their experience conducting economic evaluations from collaborating on one project with a health economist (with minimal previous exposure to economic evaluations) to leading cost analyses. Health economists’ experience conducting implementation research included evaluations of community-implemented interventions and conducting economic evaluations of implementation strategies. Although not all health economists were specifically engaged in measuring implementation costs, each had participated in research that shared the objectives of implementation research, including enhancing the uptake of EBPs in community settings. The majority of participants were female (62.5%) and ranged in age from 30 to 77 (*M* = 46.81, *SD =* 11.64). All participants had doctoral degrees and ranged from early career to established, senior researchers.

### Data collection procedures

Participants completed a brief demographic questionnaire and a semi-structured interview conducted by either the first or second authors. Interviewers were early career implementation researchers, with doctorates and faculty appointments in clinical psychology, who were interested in cross-disciplinary research with health economists. Some participants knew the interviewers through academic societies, conference attendance, or overlapping research interests. To build rapport with all participants, reasons for doing the study were explained at the onset of the interview. Interviews were conducted and recorded via Zoom and lasted from 13tes to 40 minutes (*M =* 25.69, *SD =* 6.91). An iterative process of interviews and analysis was conducted, in which the interviewers had regular meetings to review notes and themes they were beginning to identify. Efforts were made to reach saturation, or the point in which data begins to repeat and additional data collection becomes redundant, which has been shown to occur with 6–12 interviews when the same questions are asked of a relatively homogenous set of respondents [21, 22]. We also sought to balance the perspectives of implementation researchers and health economists. The research team determined that saturation had been reached following 16 interviews (8 implementation researchers and health economists respectively) due to redundancy in interview content. Participants were offered a $20 gift card in exchange for completion of the interview. Participants were informed that direct quotes only would be used in publication with permission in order to provide additional privacy protection for interviews that address experiences within academic disciplines. Only minor edits to grammar were made by participants to enhance readability.

### Measures

#### Demographic questionnaire

Participants completed short surveys with demographic and professional information, including their age, gender, race/ethnicity, degree, and primary discipline (e.g., economics, public health, psychology).

#### Semi-structured interview guide

The interview guide was developed by the research team and informed by the literature on team science, with questions derived from key factors thought to facilitate increasing capacity for interdisciplinary collaborations (e.g., conceptual issues, methodological issues, measures of success) [14, 23]. Interviews began with a general question about the participant’s research interests and the extent of their experience conducting interdisciplinary research. Subsequent questions addressed the participant’s experiences conducting interdisciplinary research with health economists and implementation researchers, challenges and facilitators to this type of research, and characteristics of these types of research partnerships. Given the purpose of this study to increase capacity for partnerships with health economists in implementation science, specific questions were asked of each discipline. Health economists were asked, “What would you like non-health economists to know about your field if they hope to partner with you?” and implementation researchers were asked, “What training would be valuable for you to increase economic methodology in your implementation research?” Interviewers asked follow-up questions as needed to clarify content and encourage elaboration. See Table 1 for comparisons between interview guides.

**Table 1:** Interview guide for health economist and implementation researchers

Topic area	Questions for health economists	Questions for implementation researchers
***Facilitators for collaborations***	What has made research partnerships with *non-health economists* successful for you?	What has made research partnerships with *health economists* successful for you?
***Barriers for collaborations***	What has led to unsuccessful research partnerships?	What has led to unsuccessful research partnerships?
***Areas for collaboration***	What components of the research are *you typically asked to partner on*?	What components of the research *do you typically ask health economists to partner on?*
	What components or types of research would you like to partner on?	Beyond partnering with health economists how have you applied economic evaluations into your implementation research?
***Timing of collaboration***	*When and how do you typically get asked to join an implementation research project*?	*How do you approach health economists to partner with you*?
***Capacity building***	*What would you like non-health economists to know about your field if they hope to partner with you*?	*What training would be valuable for you to increase economic methodology into your implementation research*?

### Data analysis

Interviews were transcribed and de-identified and uploaded into NVivo 11 Software, for coding and theme development. The data were analyzed using thematic analysis [24]. Inductive and deductive codes were first developed from the interview guides and key statements and phrases that were identified in the interviews. Then, a coding team reviewed and applied the codes across each interview, codes were clustered to develop themes, and themes were reviewed and refined. The coding team consisted of the first author and third author, along with an undergraduate research assistant. The coding team met regularly throughout the coding process to establish consensus, review discrepancies in coding, and iteratively develop the code book. The official code book was developed and revised through an iterative process until all coders reached consensus on four interviews, which included two interviews with health economists and implementation researchers respectively. Of the remaining interviews, 75% were double coded and discrepancies were resolved through discussion and code clarification.

Qualitative themes were identified and developed by looking at the convergence and comparison of codes across the two disciplines of interview participants. Following the steps outlined by Morgan (1993) regarding comparing across groups, first code occurrence across the two disciplines was used to first identify patterns in differences and similarities in themes, then text from these codes was further analyzed to interpret these patterns. Whereas the initial counts allowed the qualitative analysis team to identify areas for further in-depth analysis, the interpretation of the text provided rationale for the shared and differing perspectives across themes [25]. After themes were initially developed, they were shared and refined with the full authorship team.

## Results

An overarching finding from the qualitative interviews was that health economists and implementation researchers had differing perspectives on collaborative research efforts, which highlighted the need for improved understanding across fields. Health economists more frequently identified methodological differences, misperceptions across fields, and different disciplinary cultures, whereas implementation researchers more often discussed differences in language (e.g., “it can be really hard to learn one another’s professional languages and lexicons and that you really do have to take the time to define terms”). Though differences in language might encapsulate some of the challenges that health economists identified, it appeared that the differing perspectives were not merely semantic.

Themes were identified and grouped into the following categories that addressed (1) motivations for research collaborations and ( 2) strategies to promote successful collaborations. See Table 2 for illustrative quotes for each theme from participants from both disciplines. Quotes in the text are annotated according to participant discipline, which is denoted by abbreviation (i.e., health economist, HE; implementation scientist, IS); category, which is denoted by number; and theme, which is denoted by letter. For example, HE1a corresponds to a quote from a health economist for category 1: motivations to collaborate, theme a: goal to impact real world issues.

**Table 2:** Themes and illustrative quotes from health economists and implementation researchers

	Health economists	Implementation researchers
***1. Motivations to collaborate across disciplines on research***		
*a. Common goal to make a real-world impact*	**HE1:** I think most health economists do have a strong interest and are into the same issues that implementation researchers are interested in, like: how can we make a real-world difference with our work?”	**IS1:** Yeah, so I think we’re learning over time that training in EBP is such a cost prohibitive activity and so a lot of what I think about in terms of implementation strategies is around how much bang can you get for your buck, especially when you are working in a system.
*b. Growth as researchers and improved the quality of the science*	**HE7:** I think those kind of dialogues that you have if you’re an ongoing collaborator with somebody, helps change your whole way of looking at things and I think to me that’s one of the values of it.	**IS1:** I was really scared of calculating cost…so having this experience is really helpful in opening my eyes towards kind of what’s out there and how impactful it can be. [it] is really important for any kind of decision making.
*c. Conducting cost analyses*	**HE3:** One thing that does tend to come up, and I think this is a common misunderstanding about economics, it’s that we all do cost-benefit analysis. I don’t do cost-benefit analysis. I don’t want to do cost-benefit analysis. [Later clarified the same was true for cost-effectiveness analyses]	**IS3:** If you’re going to look at new estimated costs to an implementation strategy and then doing a cost effectiveness of one implementation strategy over another, it’s important to have the health economist involved or consulting.
*d. Relationships and shared interests*	**HE2:** Many more requests are usually for cost and cost effectiveness, and cost benefit. …economists don’t usually like doing that, so I really only agree to do it if … I really enjoy working with the partner, I know the partner. …if somebody wants me to do cost effectiveness analysis of an intervention, first of all, I have to be really interested in the intervention, the population they’re serving, what they’re trying to do. That needs to be of interest.	**IS8:** So part of it is being really inherently interested [in cost], and learning about it enough to be able to engage the economists in essentially thinking together about how they can use their tremendous vitas and skills and grant funding for all of the research that they’ve already developed, in area in which they’re already well established, to lend some of that to a collaboration in an area that may or may not be inherently interesting to them; and, to figure out how to try to make it interesting to them. And so I saw that as part of my responsibility, that was on me.
*e. Limitations of grants and publications*	**HE5:** If you’re interested in implementation science you’re never going to get into the quarterly journal of economics, it's just not gonna happen. And they don’t even really value a JAMA piece. Whereas if you’re on the medical school, that is currency.	**IS6:** I feel like the bigger challenge is, there just aren’t enough of them to go around. I know that I could ask [name of health economist] that he’ll tell me, “I’m 175% funded”
***2. Strategies to promote successful collaborations***		
*a. Timing and scope of involvement*	**HE5:** Yea, I think some people are just really good about developing partnerships early, and getting people’s input early. So instead of coming to an investigator; and it could be an economist it could be a statistician; before the grant is due and think oh, we need your help, can we put you down for five percent and can you give us a power analysis or can you help us refine the third aim. I think the most successful partnerships are one where you’ve got a complicated problem and you pull the team together early.	**IS3:** I ask [health economists] to partner on all aspects of the research and so when we’ve written grant applications, they’re part of the study team and they’ve participated, for example the latest R01 that we’ve just received, they were on the study team from the beginning to help craft the aims. So, it’s not like we just write the grants and say hey, put in an economics aim, you know, we want them to be part of it from the beginning.
*b. Methodological contributions beyond economic evaluations*	**HE2:** For those ongoing partners where we go from one project to the next to the next, they do tend to use my skillset in a broader fashion that, to involve me in thinking about the study design, and the message and kind of in involving me in the overall meetings and you know, just trying to involve me in everything really, just to be an integral part of the team. Because they understand that I have a much broader skillset than just the cost piece. I think the thing that we’re pretty good at is attacking the question…we’re really trying to push the question. What we’re interested in, which is to say is it a causal model, do we really understand causality.	**IS2:** I think that the health economists that we’ve worked with have been really methodologically rigorous and have really been involved in health services research for quite a while, so they really have a valuable perspective on the broader application as well, and, so that’s been really helpful because they’ve had input in other areas besides the cost analyses that’s been really valuable.
*c. Team Science*	**HE6:** I think one of the things that always leads to a good partnership is individuals understanding what they know and don’t know, and, of course, always having that back-and-forth that constructive-type criticism--not necessarily trying to interfere with and dictate what you should be doing—willing to work with you and learn from you.	**IS4:** I am a firm believer that you don’t need to be an expert in everything and there’s a reason why we have interdisciplinary teams. But I do think it’s helpful if you have a basic understanding of what economic evaluations look for, not at the equation level but at the broad, kind of, here are the methods, here are the types of questions you can answer.

Participant numbers included for quotes

*HE* health economist participants, *IS* implementation scientist participants

### Motivations to conduct collaborative implementation research

Health economists and implementation researchers both identified that they have a *common goal in research to impact real world issues, including policy*. These shared interests were identified as motivators to conduct collaborative research together (HE1a; IS1a). Furthermore, health economists and implementation researchers identified that *conducting collaborative research allowed for personal growth as researchers and improved the quality of the science* they conducted (HE1b; IS1b). However, *interest in collaborating on economic evaluations related to costs* differed significantly across disciplines. Implementation researchers primarily discussed partnering with health economists to conduct cost analyses, including measuring implementation costs, cost-benefit analyses, and cost-effectiveness analyses (IS1c). Although implementation researchers saw measuring costs as a primary motivation to collaborate with health economists, the majority of health economists identified that a common misperception of their field was that their research primarily focused on costs (HE1c).

In order to increase motivation for health economists to partner on cost analyses**,** health economists identified the importance of having a *strong relationship* and *a shared topic of interest* with the implementation scientist (HE1d). Though it was not recognized consistently in interviews, some implementation researchers recognized their role in motivating health economists to join projects by working with them to identify areas of interest (IS1d). *Disciplinary cultures* also contributed to differing perceptions of benefits or motivations for collaborative research. Whereas implementation researchers identified the importance of having health economists included as investigators to have the expertise needed for grant funding, implementation researchers and health economists recognized that salary support was not always beneficial for health economists, because they were either located within hard money positions or were already funded on multiple grants (IS1e; HE1e). Furthermore, health economists noted the importance of publishing in specific economic journals for researchers employed within traditional economics departments (i.e., as opposed to within schools of medicine or public health), regardless of the impact of a journal in another field (HE1e). Therefore, only certain opportunities for publications and grant funding might serve as an adequate incentive for collaboration for health economists.

### Strategies to promote effective collaborations

Researchers across disciplines identified that the *strongest collaborations occur when partnerships begin early and involve health economists in the larger scope of the project* **(**IS2a; HE2a). Health economists identified challenges in instances when they were not included fully enough or provided with the necessary resources needed to conduct rigorous economic evaluations. Researchers from both disciplines identified that *health economists have strong training in methodology* and it would be beneficial to include them in research design (IS2b; HE2b). Health economists also noted other methodological strengths, beyond cost analyses, that could contribute to implementation science, including a focus on establishing causality (HE2b) and analyses using large administrative claims datasets. In general, it was acknowledged that collaborations were most successful when there was *mutual respect across disciplines for areas of expertise* (HE2c). Though some implementation researchers had experience and expertise leading economic evaluations, the majority did not. They did, however, identify that it would be helpful to *receive training in how to lead interdisciplinary teams and increase basic understanding of health economics* (IS2c).

## Discussion

Health economics and implementation science are two fields that are situated to study real-world issues, providing a fertile landscape for collaboration to improve the public health impact of EBPs. In fact, multiple researchers that straddle the fields of health economics and implementation science have called for these collaborations to improve the rigor and understanding of the economic impact of different implementation strategies and efforts [5, 8, 26]. Both health economists and implementation researchers identify a desire to impact real-world practice and policy as a key reasons to collaborate. However, our findings also highlight potential reasons why there has been a dearth of economic research within implementation science. One of the most notable mismatches across fields was the need expressed by implementation researchers to measure costs associated with implementation strategies, and the health economists’ perspective of a common misperception of their field being a focus on calculating costs. Although there are health economists who do focus on cost analyses as their main area of research, many do not. For example, several of the health economists focused their research on evaluating the impact of health policy changes using large administrative claims data. A clear theme emerged suggesting the need to increase understanding of interests and skills across the two fields in order to foster successful collaborations.

Challenges also were noted regarding the field’s capacity regarding individuals who can conduct economic evaluations within implementation studies. Furthermore, implementation researchers and health economists discussed collaborations that would continue over multiple projects, posing additional challenges for individuals first trying to start a collaboration. As Norton and colleagues [11] noted, the most important priority amongst researchers conducting implementation science was working with someone with whom they had previously worked, which can lead to a phenomenon where the “rich get richer.” This might further exacerbate a limited workforce of health economists interested in partnering on studies in which they are primarily brought on to measure implementation costs. Two potential solutions might be needed to address these workforce issues. First, by fully engaging the expertise of health economists within implementation science, collaborations may increase and improve, providing opportunities to advance a truly transdisciplinary field focused on increasing the implementation and sustainment of EBPs. Secondly, strategies might be needed to increase the capacity of individuals who are able to conduct economic evaluations within implementation science to address the pressing need to be able to measure implementation costs, such as including master’s-level economists on projects or individuals not traditionally trained as health economists (e.g., implementation researchers with specialized training in economic evaluations).

One potential innovation could be the creation of consultation services focused on conducting economic evaluations within implementation research, including measuring implementation costs. For example, the QUERI program in the VA, which conducts implementation research, has a dedicated group of health economists to provide consultation on studies [27]. Furthermore, NIDA recently funded a multi-institutional Center of Excellence, the Center for Health Economics of Treatment Interventions for Substance Use Disorders, HCV, and HIV (CHERISH) [16]. As part of CHERISH and to address the shortage of health economics expertise, an economic consultation service was funded, which is free to researchers. Initial evaluation of CHERISH found that limiting health-economist consultation service to 6 h was feasible to support the preparation of a grant application, and often led to future collaborations with the consultant [16]. Additionally, lessons can be taken from the multiple training programs developed to build capacity in implementation science, which often lead to increased collaborations amongst the expert faculty and trainees in the programs [28, 29]. There is potential for cross-disciplinary training that aims to further integrate economists into training programs to foster collaborations and advance economic methodology in implementation science.

Our study identified the importance of effective communication and leadership across cross-disciplinary teams with health economists. These findings were consistent with a past study, which found that successful team science in implementation research was fostered when there were clear expectations and roles in conducting research, effective communication was promoted and modeled, and there was a shared goal and mission across team members [15]. Indeed, implementation researchers are frequently tasked with forming and leading large teams, which include community practitioners and researchers from multiple disciplines such as medicine, engineering, biostatistics, psychology, social work, anthropology, and economics. As opposed to learning each of these fields, implementation researchers can focus on gaining expertise in best practices in team science [14, 15, 23]. Many of the skills that facilitate other research partnerships can help to promote successful collaborations with health economists, including learning the “language” used in the field and clarifying that there is a shared understanding across researchers in terms of roles, expectations, and incentives. This overarching finding regarding team science can be expanded with three key recommendations for implementation researchers who seek to partner with health economists, which were identified in this study:

1. Identify and build relationships with health economists who seek to make a real-world impact in a shared area of research interest (e.g., substance use treatment) to promote synergy and motivation across the research team.
2. Similar to past recommendations, consultation with a health economist should occur early and include integration into planning the design and methods of the study [16]
3. Learn from health economists about other methodological strengths to advance the field of implementation science. Beyond partnering on traditional economic evaluations, the field of implementation science may benefit from additional areas of expertise that health economists offer, including a focus on determining causality within real-world settings and methodological expertise with large administrative claims data [6]. For example, one recent example of economist-led implementation research concerns the use of discrete choice experiments to define and measure how stakeholders value different implementation strategies and outcomes [30].

### Strengths and limitations

Using criterion-i and snowball sampling, we only interviewed individuals who had already conducted collaborative research within the fields of implementation science and health economists. Thus, this sample may be more interested in building collaborations and conducting team science than other implementation researchers and economists who have not yet pursued such collaborative work. These interviews provided rich insights into facilitators and barriers to existing collaborations. However, additional insights may be gained from researchers that aim to collaborate across health economics and implementation science, but have yet to secure such partnerships. Additionally, our sample of interviewees was drawn from the USA, which could limit generalizability to other contexts where the institutional structures and processes involved in health economics, implementation research, and interdisciplinary collaboration might differ. Furthermore, the interview guides focused predominately on how implementation researchers could increase capacity in economic evaluations either in their own training or by partnering with health economists. We did not systematically collect information on training needs for health economists in implementation science; however, this could be an important direction for increasing cross-disciplinary collaborations. Given qualitative findings regarding differences in methodological approaches and terminology used, it would also be beneficial to more systematically assess the understanding health economists have of implementation science objectives and concepts, and correspondingly implementation researchers have of health economics. These findings could inform training programs across the two disciplines.

Finally, this study looked specifically at how collaboration can be enhanced by strengthening the relationships between implementation researchers and health economists, while many other stakeholders participate in implementation research and practice. It is important to recognize the importance of fostering successful collaborations across various professionals, who could be part of multidisciplinary teams to conduct implementation efforts or research studies. For example, it has been recommended that clinicians collaborate directly with health economists on the establishment of clinical guidelines [31]. Furthermore, by fostering collaborations with developers of interventions and clinical guidelines, health economists, and implementation researchers, costs associated with the practice and implementation strategies could be evaluated more efficiently [32]. Future efforts should explore how to enhance collaborations across multiple fields to improve implementation efforts.

## Conclusions

In sum, our findings point to potential strategies to foster collaborations between health economists and implementation researchers and expand capacity in the field. Although differences in training, disciplinary cultures, and methodological approaches can pose challenges to these collaborations, both health economists and implementation researchers have a shared desire to partner with other disciplines involved in real-world research. With the increased interest within the implementation science field to move beyond costing of implementation strategies, and to consider the more nuanced and impactful role that economics play in large scale-up and policy, this is a serendipitous moment that should not be ignored. As implementation science increases its traction, particularly related to healthcare, economic evaluations are essential to establish feasible plans. Pragmatically, a “best-practice” implementation approach cannot be used without understanding of (a) the resources needed to execute it, (b) the consequences to the programs currently utilizing those resources, and (c) the ultimate benefit of allocating resources to the implementation. Thus, without the inclusion of economics within the scope of implementation science, the field runs the risk of perpetuating the very reason for its development—evidence-based implementation approaches will remain unused for the successful scale-up of EBPs.

## Supplementary information

**Additional file 1.** COREQ (COnsolidated criteria for REporting Qualitative research) Checklist

## Data Availability

The interviews generated and analyzed during the current study are not publicly available to protect the identities of participants, who are members of the academic community, but are available from the corresponding author on reasonable request.
